# Development and evaluation of a real‐time polymerase chain reaction assay for the rapid detection of *Talaromyces marneffei MP1* gene in human plasma

**DOI:** 10.1111/myc.12530

**Published:** 2016-07-25

**Authors:** Ha Thuc Ai Hien, Tran Tan Thanh, Nguyen Thi Mai Thu, Ashley Nguyen, Nguyen Tat Thanh, Nguyen Phu Huong Lan, Cameron Simmons, Cecilia Shikuma, Nguyen Van Vinh Chau, Guy Thwaites, Thuy Le

**Affiliations:** ^1^ Wellcome Trust Major Overseas ProgramOxford University Clinical Research UnitHo Chi Minh CityVietnam; ^2^ Hospital for Tropical DiseasesHo Chi Minh CityVietnam; ^3^ Department of Microbiology and ImmunologyPeter Doherty Institute for Infection and ImmunityUniversity of MelbourneMelbourneVICAustralia; ^4^ Hawaii Center for AIDSUniversity of Hawaii at ManoaHonoluluHIUSA; ^5^ Centre for Tropical MedicineNuffield Department of Clinical MedicineOxford UniversityOxfordUK

**Keywords:** TaqMan real‐time polymerase chain reaction, *Talaromyces marneffei*, *Penicillium marneffei*, HIV

## Abstract

Penicilliosis caused by *Talaromyces marneffei* is a common AIDS‐defining illness in South and Southeast Asia. Diagnosis is based on culture which can take up to 14 days for identification, leading to treatment delay and increased mortality. We developed a TaqMan real‐time PCR assay targeting the *MP1* gene encoding an abundant cell wall protein specific *to T. marneffei*. The assay's performance was evaluated in *MP1*‐containing plasmids, clinical isolates, and plasma from HIV‐infected patients with and without penicilliosis. The assay consistently detected 10 copies of *MP1*‐containing plasmids per reaction and 100 *T. marneffei* yeast cells per millilitre plasma. There were no amplification with seven other *Penicillium* species and six other HIV‐associated fungal pathogens tested. The assay was evaluated in 70 patients with AIDS: 50 patients with culture‐confirmed penicilliosis and 20 patients with opportunistic infections other than penicilliosis. The diagnostic sensitivity was 70.4% (19/27, 95% CI: 51.5–84.1%) and 52.2% (12/23, 95% CI: 33.0–70.8%) in plasma samples collected prior to and within 48 h of antifungal therapy respectively. The diagnostic specificity was 100% (20/20, 95% CI: 83.9–100%). This assay provides a useful tool for the rapid diagnosis of *T. marneffei* infection and has the potential to improve the management of patients with penicilliosis.

## Introduction

*Talaromyces marneffei* (previously named *Penicillium marneffei*) is an emerging fungal pathogen that causes a systemic mycosis in immunocompromised and, less commonly, immunocompetent residents and travellers to Southeast Asia, India and China.^1^, ^2^, ^3^ The HIV epidemic has transformed *T. marneffei* from a rare infection to a leading AIDS‐defining diagnosis in this region,^1^, ^4^, ^5^, ^6^, ^7^, ^8^ trailing only tuberculosis, cryptococcosis or *Pneumocystis jiroveci* pneumonia (PJP) in incidence.^1^, ^7^ Mortality is up to 20% despite antifungal therapy.^5^, ^8^ The diagnosis is often delayed as it can take up to 14 days to isolate and identify the pathogen from clinical specimens. Typical skin lesions develop in 50% to 70% of patients^1^, ^5^ which enable a presumptive diagnosis to be made based on Gram's or Giemsa staining of skin smear. Patients without skin lesions experience a significant delay in antifungal therapy initiation.^5^ These patients have significantly higher mortality and frequently die before the diagnosis is made.^1^, ^5^ Development of non‐culture‐based assays for rapid detection of *T. marneffei* infection has the potential to improve treatment outcomes.

Several serological assays detecting antigen and antibody to *T. marneffei* have been developed^9^, ^10^, ^11^; however, none has been evaluated in prospective clinical studies to diagnose active infections. Polymerase chain reaction (PCR) technique offers a sensitive and specific tool to detect several invasive fungal pathogens.^12^, ^13^ PCR‐based assays have been developed to detect *T. marneffei* in clinical isolates^14^, ^15^ and in a small number of clinical specimens.^16^, ^17^ TaqMan and SYBR Green real‐time PCR assays evaluated in 20 and 23 patients with penicilliosis reported diagnostic sensitivities of 60% and 77% respectively; however, diagnostic specificity was not assessed.^17^, ^18^ To date, the molecular targets of these PCR assays are based on the fungal ribosomal DNA, a highly conserved region shared by many groups of fungi, potentially compromising specificity. Here, we report the development and assessment of a TaqMan real‐time PCR assay to detect *T. marneffei MP1*, a novel gene encoding *T. marneffei* cell wall manoprotein.^19^ *MP1* is a unique gene without homologues in sequence databases, thus cross‐reaction to related fungal pathogens is minimised.

## Methods

### Statement of ethics

The study was approved by the Scientific and Ethical committee of the HTD. All patients gave informed consent for blood drawn for this diagnostic study.

### Development of the real‐time PCR assay

#### Oligonucleotide primers and hybridisation probe

All 23 nucleotide sequences of *T. marneffei MP1* (accession number DQ08822 to DQ08845) were retrieved from GenBank and were aligned using bioedit software (v7.0.1, Ibis Biosciences, Carlsbad, CA, USA). The primers and TaqMan probes were designed to be highly specific to a conserved region of *MP1* using primer express software (v2.0, Applied Biosystem, Foster City, CA, USA) (Table 1). The specificity of the primers and probe was confirmed by performing BLAST searches on GenBank showing no cross‐reacting sequences with other fungi.

**Table 1:** Primers and probe for the real‐time PCR assay

Primers and probe	Nucleotide sequences^a^	Position of DQ08822	Tm (°C)
Forward primer	5′‐TCTGGACGGYGTTCAGTC‐3′	222–239	57
Reverse primer	3′‐TGATTGCTTAAATCCTGAACA‐5′	294–314	52
Probe	5′‐FAM‐AAAATGAGCCTCCGCTTA GCTCCATGG‐BHQ1‐3′	249–271	52

a Y = T or C; 5′‐FAM = 5′ 6‐carboxyfluorescein; BHQ, black hole quencher.

#### Template DNA preparation

*Talaromyces marneffei* yeast cells were sub‐cultured on Sabouraud Dextrose Agar (SDA) at 37 °C for 5–7 days until single cells were created. The cells were homogenised in 5 ml PBS (pH 7.2) containing Tween 20 (5%) (Sigma, Singapore). Cell pellets were collected by centrifugation at 8000 ***g*** for 5 min, and after thoroughly washed two times with PBS, cell pellets were resuspended in 5 ml PBS. Fungal DNA extraction was performed as described previously with minor modifications.^18^ Briefly, fungal cell wall was removed to form spheroplasts by treating 200 μl of resuspended cultured fungal isolates or plasma specimens with 600 μl sorbitol buffer (1 mol l^−1^ sorbitol, 100 mmol l^−1^ EDTA and 14 mmol l^−1^ β mercaptoethanol) supplemented with 200 U lyticase enzyme (Sigma). The mixture was incubated at 30 °C for 30 min. Spheroplast DNA was extracted using QIAamp DNA blood mini kit (QIAGEN, Hilden, Germany) and was eluted in 30 μl of elution buffer.

#### TaqMan real‐time PCR assay

Real‐time PCR was performed using the LightCycler^®^480 (Roche Applied Science, Penzberg, Germany). The reaction was conducted in 25 μl volume containing 2.5 μl buffer 2.5× (QIAGEN), 500 nm MgCl_2_ (QIAGEN), 400 nm dNTP (Invitrogen), 400 nm each primer, 200 nm TaqMan probe, one unit Hotstart Taq polymerase (QIAGEN), and 5 μl template DNA. The real‐time PCR conditions were: one cycle 95 °C 15 min, 45 cycles 95 °C 15 s, 56 °C 30 s and 72 °C 15 s. The fluorescent signal of 6‐carboxyfluorescein (FAM) was detected at 72 °C in every reaction cycle. All reactions were performed in triplicate.

### Assessment of sensitivity using *T. marneffei MP1*‐containing plasmids

The real‐time PCR products were purified using the QIAquick^®^ PCR purification kit (QIAGEN, Hidden, Germany), were cloned into pCR2.1‐TOPO plasmid and electro‐transformed into *E. coli* DH5α^TM^‐T1^R^ (TOPO TA cloning kit, Invitrogen, Carlsbad, CA, USA). The presence of *MP1* gene was confirmed by sequencing using ABI BigDye Terminate v3.1 Cycle Sequencing kit (Applied Biosystem). Successful transformants were selected by culturing *E. coli* clones overnight in liquid Luria‐Bertani medium supplemented with ampicillin 100 mg ml^−1^. Plasmids were purified using QIAprep^®^ Miniprep Kit (QIAGEN), and plasmid quantity was determined using UV‐spectrophometer. To assess the analytical sensitivity of the TaqMan‐PCR assay, 10‐fold serial dilutions of purified plasmid DNA (10^6^ to 1 copies μl^−1^) in sterile distilled water were tested in triplicate.

### Assessment of sensitivity using *T. marneffei* yeast cells

The sub‐cultured *T. marneffei* yeast cells prepared as above were counted on a cell counting chamber under a light microscope. Ten‐fold serial dilutions of *T. marneffei* yeast cells (10^6^–10^1^ cells ml^−1^) spiked into human plasma collected from healthy volunteers were tested in the PCR assay. A quantity of 200 μl of plasma of each dilution was used for DNA extraction.

### Assessment of sensitivity and specificity using fungal isolates

Assessment of sensitivity and specificity was performed using seven clinical *T. marneffei* isolates and six clinically related other fungal isolates, including *Candida albicans, Candida tropicalis, Cryptococcus neoformans, Aspergillus fumigatus, Aspergillus terreus* and *Aspergillus flavus*. These clinical isolates were obtained from HIV‐infected patients at the Hospital for Tropical Disease (HTD) in Ho Chi Minh City. In addition, seven American Type Culture Collection (ATCC) laboratory *Penicillium* strains, including *P. chrysogenum* (ATCC 10106)*, P. variabile* (ATCC 32333)*, P. aurantiogriseum* (ATCC 16025)*, P. citrinium* (ATCC 10499)*, P. crustosum* (ATCC 10122)*, P. expansum* (ATCC 1117) and *P. glabrum* (ATCC 10444) were used. The fungal isolates were sub‐cultured using SDA for 3–7 days at 37 °C for the yeast isolates (*T. marneffei, Candida* spp*.,* and *Cryptococus neoformans*) and at 25 °C for the mould isolates (*Aspergillus* spp. and *Penicillium* spp.).

### Assessment of the Taqman assay in plasma samples

**Figure 1:**
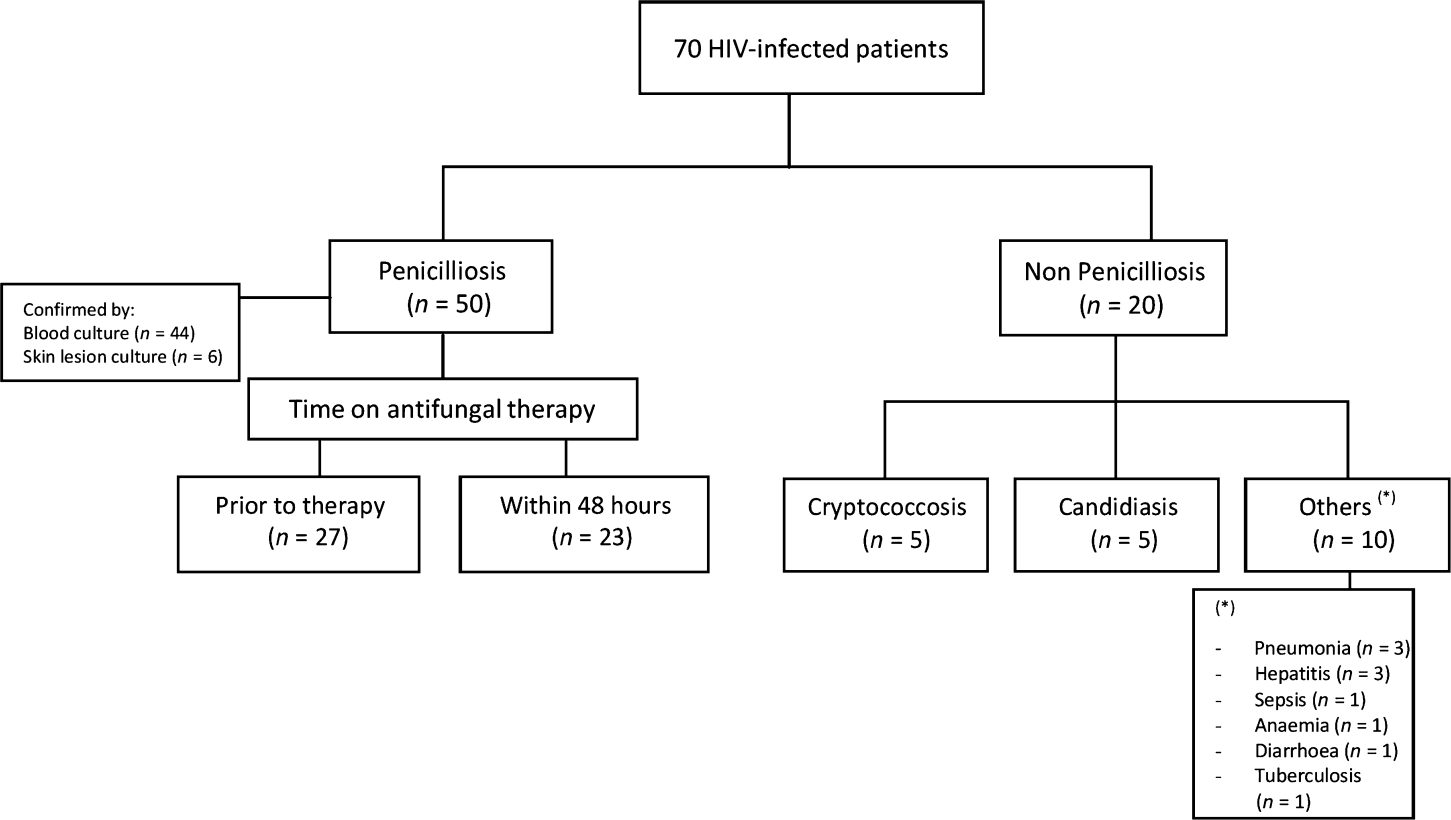
Study population used to assess the real‐time PCR diagnostic performance.

The diagnostic performance of the assay was assessed using plasma samples of HIV‐infected patients, 50 with culture‐confirmed penicilliosis and 20 with other opportunistic infections (Fig. 1). The samples came from patients who participated in a case–control study to investigate the risk factors of AIDS‐associated penicilliosis between January 2011 and July 2012 at the HTD. Blood culture was performed in all patients using the automated Bactec^™^ 9240 culture system (Becton Dickinson, Sparks, MD, USA). Penicilliosis case patients were those with an illness in which *T. marneffei* was isolated from a clinical specimen, including blood, skin scrapings, bone marrow, lymph node and/or other body fluids. Control patients were diagnosed with AIDS‐associated opportunistic infections other than penicilliosis. Plasma samples for PCR testing were collected at enrolment either prior to or within the first 48 h of antifungal therapy.

### Statement of safety

Clinical materials presumed to be infected with *T. marneffei* are handled and processed according to Biosafety Level 2 (BSL‐2) practices. All culture materials, whether in the yeast or the mould form, are handled in a Class II biosafety cabinet. All culture containment materials are covered with paraffin paper when transferred outside of the safety cabinet.

## Results

### Sensitivity for detecting *MP1* gene copy number

**Figure 2:**
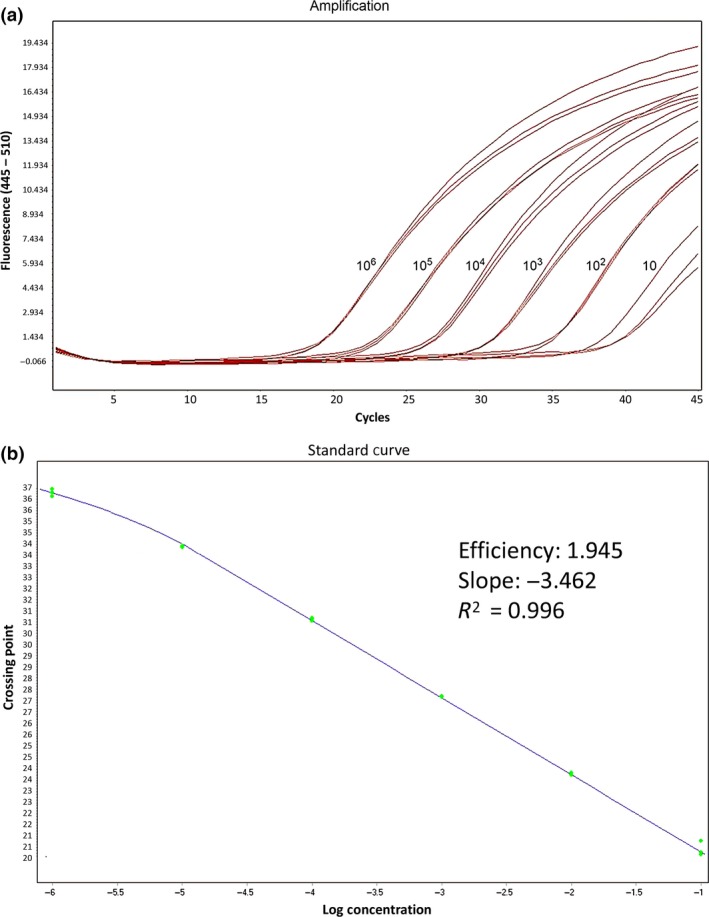
(a) Amplification plots of Mp1 plasmids with dilution concentrations from 10^6^ to 10^1^ copies per reaction, showing increase in the fluorescence emission of the reporter dye relative to the reference dye. (b) Linear regression of the crossing point values vs. log_10_ concentration of Mp1 plasmids. All the samples were performed in triplicate.

The TaqMan‐PCR assay was able to detect *MP1*‐containing plasmids at one copy per reaction (or 30 copies ml^−1^), but detection was consistent at 10 copies per reaction (or 300 copies ml^−1^). Figure 2 shows the quantification of *MP1*‐containing plasmids that were serially diluted to concentrations from 10^6^ to 10^1^ and the linear regression of the Cp values vs. the log_10_ concentrations of *MP1* plasmids. Linearity was achieved within a range plasmid DNA from 10^6^ to 10^1^ copies per reaction, and the correlation coefficient *R* ^2^ = 0.996.

### Sensitivity for detecting *T. marneffei* yeast spiked into human plasma

The TaqMan‐PCR assay was able to detect *T. marneffei* yeast cells at a concentration of 100 cells ml^−1^. Linearity was achieved within a range of yeast concentrations from 10^6^ to 10^2^ cells ml^−1^, *R* ^2^ = 0.997. The calculated number of plasmids at the limit of detection of 100 cells ml^−1^ is 30 copies ml^−1^, indicating significant DNA loss during extraction.

### Specificity for detection of *T. marneffei* among fungal isolates

The primers and probe successfully detected all seven *T. marneffei* clinical isolates. No significant elevated signal was detected in the 13 non‐*T. marneffei* isolates. The absence of DNA was confirmed by agarose gel electrophoresis of TaqMan‐PCR products derived from these non‐*T. marneffei* isolates, indicating that the assay is highly specific.

### Diagnostic performance in patient plasma

The assay was evaluated in plasma samples from 50 HIV‐infected patients with culture‐confirmed penicilliosis and from 20 patients with other HIV‐associated opportunistic infections. The assay was able to detect *T. marneffei MP1* gene in 19 of 27 (70.4%, 95% CI: 51.5–84.1%) patients in whom samples were collected prior to antifungal therapy and in 12 of 23 (52.2%, 95% CI: 33.0–70.8%) patients in whom samples were collected within 48 h of antifungal therapy. Although sensitivity is lower compared to the Bactec system (44/50 patients, 88.0%, 95% CI: 76.2–94.3%), a statistical comparison between the sensitivity of the new TaqMan PCR assay and the Bactec system in the patients where both samples were collected prior to antifungal therapy did not reach significance, 19/27 (70.4%) vs. 24/27 (88.9%), *P* value (McNemar test) = 0.29. No visible signal was detected in all 20 plasma specimens of patients infected with other opportunistic infections, giving a clinical specificity of 100%.

Table 2 shows the Cp values in the 50 plasma samples of the patients with culture‐confirmed penicilliosis, timing of blood collection with respect to antifungal therapy, and the duration of the Bactec culture system to detect fungal growth from the hospital microbiology laboratory. The median time to detect a growth signal in blood culture bottles using the Bactec system was 5 days (inter quartile range: 5–7). Among the 16 patients who did not have skin lesions, the PCR assay was positive in only seven (43.8%); however, sensitivity was higher, 66.7%, when blood samples were collected prior to antifungal therapy (Table 2).

**Table 2:** TaqMan real‐time PCR detection of *T. marneffei MP1* gene in 50 plasma samples of patients with microbiological‐confirmed penicilliosis

Patients	Time on antifungal therapy (h)^a^	Mean Cp values (standard deviation)	Specimens positive with *T. marneffei*	Time to Bactec blood culture identification (days)^b^
1	0	39.5 (±0.95)	Blood and skin	9
2	0	40.0 (±0.00)	Skin	Data not available
3	0	34.4 (±0.05)	Blood and skin	Data not available
4	0	40.0 (±0.00)	Skin	Data not available
5	0	35.7 (±0.97)	Blood	3
6	0	Not detected	Skin	Data not available
7	0	40.0 (±0.00)	Blood and skin	4
8	0	Not detected	Blood	6
9	0	Not detected	Blood and skin	7
10	0	39.0 (±1.74)	Blood and skin	6
11	0	38.6 (±0.67)	Blood and skin	3
12	0	35.0 (±0.24)	Blood and skin	4
13	0	38.1 (±1.25)	Blood and skin	5
14	0	Not detected	Blood	Data not available
15	0	39.3 (±0.63)	Blood and skin	4
16	0	34.0 (±0.34)	Blood	Data not available
17	0	Not detected	Blood and skin	5
18	0	39.6 (±0.62)	Blood	6
19	0	35.2 (±0.11)	Blood and skin	5
20	0	Not detected	Blood and skin	Data not available
21	0	40.0 (±0.00)	Blood and skin	7
22	0	Not detected	Blood and skin	3
23	0	Not detected	Blood and skin	7
24	0	40.0 (±0.00)	Blood and skin	5
25	0	40.0 (±0.00)	Blood and skin	7
26	0	40.0 (±0.00)	Blood and skin	5
27	0	33.0 (±1.25)	Blood	6
28	24	Not detected	Skin	Data not available
29	24	Not detected	Blood and skin	7
30	24	38.2 (±0.23)	Blood and skin	5
31	24	37.2 (±0.10)	Blood and skin	3
32	24	Not detected	Skin	Contaminate (B*urkholderia cepacia*)
33	24	Not detected	Blood	6
34	24	40.0 (±0.00)	Blood and skin	4
35	24	40.0 (±0.00)	Blood and skin	6
36	24	Not detected	Blood	5
37	24	40.0 (±0.00)	Blood and skin	9
38	24	40.0 (±0.00)	Blood	5
39	24	Not detected	Blood	7
40	24	Not detected	Blood	5
41	24	31.0 (±1.20)	Blood and skin	3
42	24	33.0 (±0.47)	Blood and skin	5
43	24	Not detected	Blood and skin	7
44	48	37.8 (±0.33)	Skin	Data not available
45	48	Not detected	Blood	Data not available
46	48	36.9 (±0.29)	Blood	7
47	48	Not detected	Blood	6
48	48	Not detected	Blood	7
49	48	39.9 (±0.24)	Blood	5
50	48	39.3 (±0.63)	Blood and skin	7

a Time on antifungal therapy prior to blood collection for real‐time PCR assay, time 0 = prior to antifungal therapy.

b Time to detect a growth signal in blood culture bottles using the automated Bactec culture system; median: 5 days (interquartile range: 5–7). All reactions were performed in triplicate.

## Discussion

We developed a highly specific PCR assay for *T. marneffei* detection with no amplification detected from the 13 different fungal isolates in the *Cryptococcal, Aspergillus, Candida* and *Penicillium* genera, and no amplification was observed in plasma specimens of 20 AIDS patients infected with other HIV‐associated fungal and opportunistic pathogens. The diagnostic sensitivity was 70.4% in samples collected prior to antifungal therapy, which decreased to 52.2% in samples collected within 48 h after antifungal drugs had been started, suggesting that the assay can be useful to monitor fungal clearance on therapy. With respect to the analytical sensitivity, the assay had a limit of detection of one copy of *MP1* per reaction, but it consistently detected 10 copies per reaction (or 300 copies per millilitre). In *T. marneffei* spiked human plasma, the assay consistently detected 100 cells ml^−1^. The analytical sensitivity of our PCR assay appears to be 10‐fold lower compared to the TaqMan real‐time PCR assay by Pornprasert *et al*. 18 which targets the 5.8S ribosomal DNA. This may mean that there are significantly more copy numbers of the ribosomal DNA relative to the *MP1* per *T. marneffei* yeast cell. However, the diagnostic sensitivities of the two assays (in samples collected before and after antifungal therapy) are similar, 31/50 (62.0%) vs. 12/20 (60.0%), *P* value (Fisher's Exact Test) = 1.00. The diagnostic sensitivity of our assay is lower than the recently developed SYBR Green real‐time PCR assay by Sha Lu *et al*.; however, the difference in sensitivities is not statistically significant, 31/50 (62.0%) vs. 23/30 (76.7%), *P* value (Fisher's Exact Test) = 0.22. In none of these previous real‐time PCR assays was clinical specificity assessed. Direct comparison of the diagnostic performance of these real‐time PCR assays on the same clinical samples will be informative. Our assay is not more sensitive than the nested PCR assay developed by Pongpom *et al*. 16 which detected *T. marneffei* in 24/35 patients (68.6%); however, conventional PCR methods are slower, more labour‐intensive, and is prone to post‐PCR contamination comparing to real‐time PCR.

Compared to the Bactec culturing method, our assay was less sensitive (70% vs. 88%), although the difference did not reach statistical significance. A major advantage of culturing is the larger volume of sample being used (approximately 5 ml of blood compared to 200 μl used in this assay). Using larger DNA extraction volume and further concentrating the DNA used in PCR reaction might increase the sensitivity of this assay.

Recently, a monoclonal‐based immunoassay has been developed that detected *T. marneffei* Mp1p antigen in patient plasma with a sensitivity of 75% (15/20) and specificity of 99.4% (537/540).^10^ The assay has been shown to detect Mp1p antigenemia in 9.4% of more than 8000 HIV‐infected patients in outpatient clinics from Guangzhou, China,^20^ offering another tool for rapid diagnosis of *T. marneffei* infection.

A limitation of our study is that we only tested the assay on patient plasma samples. Whole blood or white blood cell buffy coat may be better diagnostic specimens as *T. marneffei* primarily replicates inside macrophages, which can be lost during plasma sample processing. Another limitation is that the Cp values of the PCR‐positive specimens in our study are high, suggesting that the amount of pathogen DNA obtained for PCR is not high enough. Research for better ways to break down the fungal cell wall using both mechanical and chemical means during DNA extraction and for ways to increase the amount of input DNA used in PCR assays may advance the molecular diagnostics of fungal diseases.

Despite the limitations and the need for further research to improve the sensitivity, the clear advantages of this real‐time PCR assay are its speed and high specificity. It takes 5–6 h to perform, which would probably result in a next‐day diagnosis in most routine laboratories. This compares to an average of 5 days to detect a growth signal in the blood culture of patients using the highly efficient Bactec system (Table 2). Additionally, identification of *T. marneffei* requires a demonstration of temperature‐dependent dimorphic growth, and subculture at 25 °C generally takes another 2–3 days. This real‐time PCR assay should not replace the need for conventional microbiology methods in diagnosing penicilliosis. However, in conjunction with culturing, it can be used as a rapid rule‐in test that can make a significant difference in patient management by allowing antifungal therapy to begin sooner, particularly in patients without skin lesions, and has the potential to improve the outcomes of *T. marneffei*‐infected patients.

## Conflicts of interest

We declare that we have no conflicts of interest.

## Funding

The study was funded by the University of Washington Centre for AIDS Research (NIH grant P30 AI027757) and the Hawaii Centre for AIDS as an International Junior Faculty Award (Le) and the Wellcome Trust (Hien, Thanh, Thu, Lan, Thwaites, and Le).

## Author contributions

Study concept and design: Hien, Thanh TT, Simmons, Le. Obtaining funding: Le, Shikuma. Clinical and microbiology data acquisition: Le, Thanh NT, Lan, Vinh Chau. Laboratory work: Hien, Thanh, Thu, Ashley. Analysis and interpretation of data: Hien, Thanh, Le. Drafting the manuscript: Hien, Thanh TT, Le. Critical revision of the paper for intellectual contents: Thu, Ashley, Thanh NT, Shikuma, Lan, Vinh Chau, Simmons, and Thwaites. All authors contributed to and approved the final manuscript.
